# Maternal Vitamin D Status and Its Associated Environmental Factors: A Cross-Sectional Study

**DOI:** 10.4314/ejhs.v32i5.3

**Published:** 2022-09

**Authors:** Woon Fui Chee, Arif Sabta Aji, Nur Indrawaty Lipoeto, Chin Yit Siew

**Affiliations:** 1 Department of Nutrition, Faculty of Medicine and Health Sciences, Universiti Putra Malaysia, Selangor, Malaysia; 2 Graduate School of Public Health, Faculty of Health Sciences, Alma Ata University, Yogyakarta, Indonesia; 3 Department of Nutrition, Faculty of Health Sciences, Alma Ata University, Yogyakarta, Indonesia; 4 Department of Nutrition, Faculty of Medicine, Andalas University, Padang, West Sumatra, Indonesia; 5 Research Centre of Excellence, Nutrition and Non-communicable Diseases, Faculty of Medicine and Health Sciences, Universiti Putra Malaysia, Selangor, Malaysia

**Keywords:** Vitamin D, pregnancy, intake, body surface area, Indonesia, Malaysia

## Abstract

**Background:**

Vitamin D deficiency is common among women during pregnancy. This study aims to determine the prevalence of vitamin D deficiency and their shared modifiable environmental factors among pregnant women in Indonesia and Malaysia.

**Methods:**

Blood samples of 844 third-trimester pregnant women (Indonesians: 311; Malaysians: 533) were collected to determine their serum 25(OH) D levels. Information on sun exposure and sun protection behaviours were obtained through face-to-face interviews. Dietary vitamin D intake was assessed by using a semiquantitative food frequency questionnaire.

**Results:**

The prevalence of vitamin D deficiency (<30 nmol/L) among Indonesian and Malaysian pregnant women were 42.4% and 72.0%, respectively. Percentage of exposed body surface area was inversely associated with vitamin D deficiency among Indonesian pregnant women (OR = 0.21, 95% CI = 0.09–0.48). Among Malaysian pregnant women, higher intakes of dietary vitamin D were associated with lower risk of vitamin D deficiency (OR = 0.48, 95% CI = 0.29–0.81). Analysis of the combined cohorts revealed a lower risk of vitamin D deficiency among pregnant women who had a daily intake of at least 15 mcg vitamin D (OR = 0.58, 95% CI = 0.38–0.88) and exposure of more than 27% body surface area to the sunlight (OR = 0.30, 95% CI = 0.16–0.60).

**Conclusions:**

Despite abundant sunshine, vitamin D deficiency is prevalent among pregnant women in tropical countries. The present study suggests that nutrition education on vitamin D intake and sun exposure during pregnancy is necessary for primary prevention of vitamin D deficiency in pregnant women living in the tropical countries.

## Introduction

Vitamin D, a fat-soluble secosteroid, has long been recognised for its importance in musculoskeletal health (1). Sun exposure is the main source of vitamin D, which is mainly influenced by seasons, duration of exposure, size of exposed body surface area, skin pigmentation, dress habits, and sunscreen use (2). Apart from sunlight, vitamin D can be obtained from foods such as oily fish (salmon, sardines and mackerel), egg yolk, cheese, beef liver, and supplements (3).

Despite the lack of consensus on an optimal vitamin D level, vitamin D inadequacy (defined as 25-hydroxyvitamin D [25(OH)D] <50 nmol/L (4)) is a universal health problem that affects populations of all ages, races, and geographical locations (5). Pregnant women are one of the vulnerable groups for vitamin D inadequacy. During pregnancy, maternal vitamin D metabolism changes significantly to attain fetal bone mineral accretion (6). Vitamin D status of the pregnant women determines the vitamin D status of her newborn infant (7). Poor vitamin D status during pregnancy could lead to adverse maternal and fetal outcomes such as preeclampsia, emergency cesarean section delivery, and premature birth (8–10). Emerging evidence over the past few years have demonstrated the important role of maternal vitamin D status during pregnancy in fetal programming, leading to several non-skeletal outcomes such as malnutrition and allergic diseases in the offspring (11,12).

Indonesia (0°N) and Malaysia (2°N) are two neighboring tropical countries located right next to the equator that receive plentiful of sunshine throughout the year. Minangkabau are the majority ethnic group in West Sumatra, Indonesia (13), while Malays are the largest ethnic group in Peninsular Malaysia (14). Both Minangkabau and Malays share the similar religious and cultural practices where their primary religion is Islam and wearing a *Hijab*, a veil covering the head and chest, and clothing that covers most part of the body except face, hands, and feet, is commonly practiced by Muslim women (15). Meanwhile, both Minangkabau and Malays shared similar physical characteristics, in which a majority of them have brown (type IV) or dark brown (type V) skin colour according to the Fitzpatrick's scale (16–18).

Despite abundance of sunlight, previous studies have revealed a widespread of vitamin D inadequacy among third-trimester pregnant women living in Indonesia and Malaysia, with prevalence range from 45.5% to 82.2% (16,17). Concealing clothing due to cultural and religion reasons reduces the exposure of skin to the sunlight and subsequently increases the risk of vitamin D inadequacy among pregnant women, despite living in tropical countries (17,19). Apart from concealing clothing, there is lack of vitamin D fortification in foods and very few natural foods have high vitamin D content in both Indonesia and Malaysia (20). Vitamin D fortification in food products such as bread, breakfast cereals, dairy products, and fruit juices in Indonesia and Malaysia are based on a voluntary basis by the food manufacturers (21).

To date, a few studies have assessed vitamin D status of pregnant women living in the tropics and it is less clear about their vitamin D intake and sun exposure behaviours (16,17,19). While population of both Indonesia and Malaysia share similar characteristics such as religion, culture, and skin colour, no published studies have investigated their shared risk factors of vitamin D deficiency. As both Indonesia and Malaysia are located right next to the equator, this provides an opportunity to assess vitamin D status in the absence of seasonal variation in ultraviolet radiation exposure. Therefore, this study aims to determine the prevalence of vitamin D deficiency and their shared modifiable environmental factors among pregnant women in tropical countries (Indonesia and Malaysia). The shared environmental factors identified in the present study can be targeted in possible future intervention strategies for primary prevention of vitamin D deficiency among pregnant women living in tropical countries.

## Materials and Methods

**Study design and subjects**: This study used data from the Vitamin D of Pregnant Mothers (VDPM) conducted between September 2016 and March 2018 and data from the Mother and Infant Cohort Study Malaysia (MICOS) conducted between November 2016 and January 2018. The design and methodology of these two studies have been described in detail elsewhere (22–24). Briefly, pregnant women with a singleton pregnancy and attending the selected public health clinics for regular antenatal check-ups were recruited during the third trimester of pregnancy (≥28 weeks of gestation). A total of 311 pregnant women from the West Sumatra province in Western Indonesia (0°N, 102°E) and 533 pregnant women from the state of Selangor (3°N, 101°E) and the Federal Territory of Kuala Lumpur (3°N, 101°E), Malaysia who completed the study were included in analyses.

**Data collection**: Information on characteristics of the respondents including age at conception, ethnicity, educational level, monthly household income, work status, and parity were obtained through a face-to-face interview. Data on pre-pregnancy body weight and height were extracted from antenatal care records. Pre-pregnancy Body Mass Index (BMI) was calculated and classified according to the World Health Organization (WHO) guidelines (25).

Maternal vitamin D intake and supplementation were assessed using a validated semi-quantitative vitamin D food frequency questionnaire specifically developed for the respondents in Indonesia (26) and Malaysia (27), respectively. Briefly, the questionnaire consists of natural foods rich in vitamin D, foods that were fortified with vitamin D, and dietary supplements that contained vitamin D. Due to non-availability of vitamin D in Indonesian and Malaysian food composition tables, the vitamin D content of natural foods was obtained from the United States Department of Agriculture National Nutrient Database for Standard Reference (USDA) (28), while vitamin D content of the fortified food products and dietary supplements was obtained from the products' label. Daily vitamin D intake was calculated and compared with the Recommended Dietary Allowance (RDA) for Indonesian pregnant women (29) and Recommended Nutrient Intakes (RNI) for Malaysian pregnant women, respectively (30). The RDA and RNI for vitamin D in Indonesian and Malaysian pregnant women were 15 mcg per day, respectively (29,30). Vitamin D intake was further classified as adequate (≥15 mcg) and inadequate (<15 mcg) according to the recommended intake.

Pregnant women were required to recall the amount of time they spent outdoors, the clothing they wore, and sunscreen used in the past week (31,32). Duration of sun exposure was calculated as the average time spent in the sun daily during their leisure and working time. As findings from a previous study suggested that Muslim women wearing the *hijab* should expose to the sunlight for at least 65 minutes per day in order to satisfy the body's requirement for vitamin D (19), the duration of sun exposure in the present study was further categorised into “<60 minutes” and “≥60 minutes”. Percentage of body surface area (BSA) exposed to sunlight was estimated by using the “Rule of Nine” (32) and categorised into “<27%” and “≥27%”, as proposed by Perampalam et al. (33) in order to induce an increase in vitamin D level.

**Laboratory analysis**: A venous blood sample of 2mL was collected from the pregnant women at third trimester during their routine antenatal check-up at the selected health clinics and gestation age at blood withdrawal was recorded. Blood samples of 311 Indonesian pregnant women were sent to the Biomedical Laboratory at the Faculty of Medicine, University of Andalas and were analysed using ELISA from Diagnostic Biochemistry Canada (DBC) 25(OH)D (DBC, London, Ontario Canada). The assay has an intra-assay coefficient of variation of 5.8% at 25.32 ng/mL and 1.39% at 17.95 ng/mL and the inter-assay coefficient of variation was 8.4% at 24.6 ng/mL and 5.52% at 24.26 ng/mL, respectively. Meanwhile, the blood samples of 533 Malaysian pregnant women were sent to the laboratory at Pantai Premier Pathology, Kuala Lumpur, Malaysia and were analysed by using the Siemens ADVIA Centaur Vitamin D Total assay (Siemens, Tarrytown, NY, USA). The assay has been standardised to the University of Ghent reference measurement procedure and has achieved the Centers for Disease Control Vitamin D Standardization Certification (34,35). The serum 25(OH)D concentrations were then classified into vitamin D deficiency (<30 nmol/L), insufficiency (<50 nmol/L) and sufficiency (≥50 nmol/L) according to the National Academy of Medicine, formerly known as Institute of Medicine (IOM) (4).

**Data analysis**: Statistical analysis was performed using IBM SPSS Statistics 22 software (IBM SPSS Armonk, NY). Due to the low number of pregnant women with sufficient vitamin D level (≥50 nmol/L), there may be insufficient statistical power to detect the significant associations between the environmental factors and maternal vitamin D status. Therefore, we decided to recategorise the vitamin D status into non-deficient (≥30 nmol/L) and deficient (<30 nmol/L) group. Associations between environmental factors and vitamin D deficiency were assessed using a multivariable generalised linear mixed model with country of origin and pregnant women entered as random effect. Vitamin D intake, intake of vitamin D supplements, duration of sun exposure, percentage of BSA exposed to the sunlight, and sunscreen application were entered as fixed effect. Vitamin D status was entered as outcome variable with the non-deficient group as reference group. Multivariable model was adjusted for potential confounding variables significantly associated with vitamin D deficiency identified from the univariable model and previous literature. Data were presented as odds ratio (OR) with 95% confidence interval (CI). The statistical significance was set at *p*<0.05.

**Ethics approval**: Study conducted in Indonesia was approved by the Research Ethics Committee of the Medical Faculty, Andalas University, West Sumatra, Indonesia (108/KEP/FK/2016), while study conducted in Malaysia was approved by the Ethics Committee for Research Involving Human Subjects, Universiti Putra Malaysia [FPSK(FR16)P006] and the Medical Research and Ethics Committee (MREC), Ministry of Health Malaysia (NMRR-16-1047-30685). Written informed consent was obtained from all respondents prior to data collection.

## Results

**Characteristics of pregnant women**: In total, 844 third-trimester pregnant women were included in the present study, of which 311 of them were Indonesians and 533 were Malaysians, respectively (Table 1). The mean daily intake of vitamin D was significantly higher among pregnant women in Malaysia (10.2±7.9 mcg) than those in Indonesia (8.5±6.1 mcg) (*p*<0.001). Similarly, the proportion of those who achieved the recommended vitamin D intake of ≥15 mcg daily was significantly higher among the Malaysian pregnant women (25.7%) as compared to the Indonesian pregnant women (13.5%) (*p*<0.001). Malaysian pregnant women (33.6%) were more likely to consume vitamin D supplements during the third trimester of pregnancy as compared to the Indonesian counterparts (24.4%) (*p*=0.005). Compared to the Malaysian pregnant women (4.5%), the Indonesian pregnant women (47.3%) spent more time outdoors and exposed to the sunlight for ≥60 minutes daily (*p*<0.001). The proportion of pregnant women who exposed ≥27% of their BSA to the sunlight (13.8% vs. 3.0%) and applied sunscreen (72.7% vs. 41.3%) were significantly higher among the Indonesian pregnant women than the Malaysian pregnant women (*p*<0.001).

**Table 1:** Characteristics of 844 third trimester pregnant women

Characteristics	Indonesian n = 311	Malaysian n = 533	*p*-value
Location, n (%)			
Rural	152 (48.9)	0	<0.001
Urban	159 (51.1)	533 (100.0)	
Age at conception, mean (SD), years	29.7 (5.9)	30.0 (4.1)	0.50
Gestational age at blood withdrawal, mean (SD), weeks	31.5 (2.8)	32.1 (3.6)	0.007
Ethnicity, n (%)			
Malay	0	492 (92.3)	<0.001
Minangkabau	294 (94.5)	0	
Others *	17 (5.5)	41 (7.7)	
Educational level, n (%)			
Primary	68 (21.9)	0	<0.001
Secondary	163 (52.4)	97 (18.2)	
Tertiary	80 (25.7)	436 (81.8)	
Monthly household income, n (%)†			
Low	101 (32.5)	91 (17.1)	<0.001
Moderate	91 (29.2)	280 (52.5)	
High	119 (38.3)	162 (30.4)	
Work status, n (%)			
Non-working	216 (69.5)	164 (30.8)	<0.001
Working	95 (30.5)	369 (69.2)	
Parity, n (%)			
Primiparous	90 (28.9)	225 (42.2)	<0.001
Multiparous Pre-pregnancy BMI, n (%), kg/m^2^	221 (71.1)	308 (57.8)	
Underweight (<18.5)	45 (14.5)	49 (9.2)	0.005
Normal weight (18.5–24.9)	180 (57.9)	287 (53.8)	
Overweight/Obesity (≥25.0)	86 (27.7)	197 (37.0)	
Mean (SD)	22.9 (4.3)	24.1 (4.9)	<0.001
Dietary vitamin D intake per day, n (%), mcg			
<15	269 (86.5)	396 (74.3)	<0.001
≥ 15	42 (13.5)	137 (25.7)	
Mean (SD)	8.5 (6.1)	10.2 (7.9)	<0.001
Intake of supplements containing vitamin D, n (%)			
No	235 (75.6)	354 (66.4)	0.005
Yes	76 (24.4)	179 (33.6)	
Duration of sun exposure per day, n (%), min			
<60	164 (52.7)	509 (95.5)	<0.001
≥60	147 (47.3)	24 (4.5)	
Median (IQR) ‡	50.0 (30.0, 75.0)	4.3 (0, 17.1)	<0.001
Body surface area exposed to sunlight per day, n (%), %			
≤27	268 (86.2)	517 (97.0)	<0.001
>27	43 (13.8)	16 (3.0)	
Sunscreen application, n (%)			
No	85 (27.3)	313 (58.7)	<0.001
Yes	226 (72.7)	220 (41.3)	

BMI - body mass index, IQR - interquartile range, SD - standard deviation

* Other ethnicities included Chinese, Indian, Javanese, and Batak.

† Monthly household income was categorised as low (<IDR2,000,000; <RM2300), moderate (IDR2,000,000-IDR3,000,000; RM2300-RM5599), and high (>IDR3,000,000; ≥RM5600). 1 US dollar = Indonesian Rupiah (IDR) 14,345 = Malaysian Ringgit (RM) 4.19 (as of Feb 14, 2022).

‡ Data presented as median (IQR) for non-normally distributed dat

**Maternal vitamin D status in third trimester of pregnancy**: The mean serum 25 (OH)D concentrations was significantly higher among Malaysian pregnant women as compared to the Indonesian pregnant women (*p*<0.001) (Table 2). Compared to Malaysian pregnant women (42.4%), Indonesian pregnant women (72.0%) had a significantly higher prevalence of vitamin D deficiency (*p*<0.001).

**Table 2:** Vitamin D status of 844 third trimester of pregnant women

Maternal Vitamin D Status, n (%) (nmol/L)	Indonesia n (%) n = 311	Malaysia n (%) n = 533	*p*-value
Deficiency (<30)	224 (72.0)	226 (42.4)	<0.001
Insufficiency (30–49.9)	79 (25.4)	264 (49.5)	
Sufficiency (≥50)	8 (2.6)	43 (8.1)	
Mean (SD)	24.4 (11.5)	33.9 (12.9)	<0.001

**Factors associated with maternal vitamin D deficiency**: Results from the adjusted multivariable model (Table 3) shows that exposure of BSA of ≥27% was associated with lowered odds of vitamin D deficiency in Indonesian pregnant women (OR=0.21, 95%CI=0.09–0.48). On the other hand, Malaysian pregnant women who had ≥15 mcg of vitamin D intake from food daily were less likely to have vitamin D deficiency (OR=0.48, 95%CI=0.29–0.81). When data from both countries were combined, results from the adjusted multivariable model indicates that the odds of having vitamin D deficiency were reduced by 42% in pregnant women who had ≥15 mcg vitamin D intake daily (OR=0.58, 95%CI=0.38–0.88). Meanwhile, pregnant women who exposed ≥27% of BSA to the sunlight were less likely to have vitamin D deficiency (OR=0.30, 95%CI=0.16–0.60).

**Table 3:** Multivariable generalized linear mixed model of environmental factors associated with maternal vitamin D deficiency

	Indonesia		Malaysia		Combined Cohorts	
	
	Crude	Adjusted *	Crude	Adjusted *	Crude	Adjusted *
	
	OR (95% CI)	OR (95% CI)	OR (95% CI)	OR (95% CI)	OR (95% CI)	OR (95% CI)
Dietary vitamin D intake per day, mcg						
<15	Reference	Reference	Reference	Reference	Reference	Reference
≥15	0.94 (0.41–2.17)	1.04 (0.44–2.47)	0.56 (0.29–1.09)	0.48 (0.29–0.81)	0.62 (0.41–0.92)	0.58 (0.38–0.88)
Intake of supplements containing vitamin D						
No	Reference	Reference	Reference	Reference	Reference	Reference
Yes	1.19 (0.61–2.33)	1.13 (0.57–2.26)	0.72 (0.39–1.30)	0.90 (0.55–1.47)	0.83 (0.58–1.19)	0.96 (0.66–1.40)
Duration of sun exposure per day, min						
<60	Reference	Reference	Reference	Reference	Reference	Reference
≥60	0.80 (0.46–1.37)	0.74 (0.41–1.35)	1.47 (0.44–4.85)	1.57 (0.62–4.00)	0.94 (0.60–1.46)	0.87 (0.54–1.40)
Body surface area exposed to sunlight per day, %						
<27	Reference	Reference	Reference	Reference	Reference	Reference
≥27	0.36 (0.18–0.74)	0.21 (0.90–0.48)	0.32 (0.06–1.87)	0.54 (0.13–2.27)	0.35 (0.19–0.62)	0.30 (0.16–0.60)
Sunscreen application						
Yes	Reference	Reference	Reference	Reference	Reference	Reference
No	0.66 (0.36–0.19)	0.74 (0.40–1.39)	0.98 (0.60–1.60)	1.10 (0.75–1.62)	0.86 (0.64–1.17)	0.97 (0.71–1.33)

OR - odds ratio, CI - confidence interval

* Model adjusted for location, age at conception, gestational age at blood withdrawal, ethnicity, educational level, work status, monthly household income, parity, and pre-pregnancy BMI

## Discussion

The present study shows that vitamin D deficiency is widespread among pregnant women living in tropical countries, with 72.0% among Indonesian pregnant women and 42.4% among Malaysian pregnant women. We found that compliance of vitamin D intake according to the dietary guidelines and exposure of larger surface areas to the sun are protective against maternal vitamin D deficiency.

In the present study, results from the separate analysis for the two countries showed that the percentage of exposed BSA was inversely associated with the risk of vitamin D deficiency among Indonesian pregnant women, while no association was found among Malaysian pregnant women. In contrast, adequate intake of dietary vitamin D was associated with lower risk of vitamin D deficiency among Malaysian pregnant women, while no association was found among Indonesian pregnant women. Our results are consistent with previous studies that the percentage of exposed BSA was significantly associated with maternal serum 25(OH)D levels among Indonesian pregnant women (17,19). Similarly, a recent study reported that maternal vitamin D intake is a protective factor against vitamin D deficiency in Malaysian pregnant women (16). In the present study, Indonesian pregnant women spent significantly more time outdoor as compared to the Malaysian pregnant women and thus the percentage of exposed BSA plays an important role in determining the amount of the sunlight that reach the skin and converts into vitamin D. On the other hand, the Malaysian pregnant women in the present study were recruited from the urban areas and sunlight is not a major source of vitamin D due to lack of involvement in outdoor activities, which is indicated by short duration of sun exposure and thus have to obtain vitamin D through dietary intake.

Contradictory to previous study (16), we found no significant associations between the intake of supplements containing vitamin D and vitamin D deficiency among Indonesian and Malaysian pregnant women. The non-significant associations may be explained by the low consumption of vitamin D containing supplements during pregnancy in both Indonesian and Malaysian women. Limited prevalence data to address the issue of vitamin D deficiency, lack of awareness on the health consequences of poor vitamin D status, and no specific recommendations on vitamin D supplementation might explain the low vitamin D supplementation in the present study (37).

Our study did not find an association between the duration of sun exposure and vitamin D deficiency in both Indonesian and Malaysian pregnant women, which supports the findings from previous studies conducted in tropical countries (17,36). The non-significant association between duration of sun exposure and vitamin D status is likely to be explained by the sun protection behaviours such as sunscreen application and concealed dress styles. Majority of the pregnant women in our study are Muslim who wear *hijab*, Islamic clothing that covers the whole body but leaves the face and hands exposed (approximately 13.5% of body surface area). A study conducted among Indonesian pregnant women suggested that women with *hijab* should expose to the sunlight at least 64.5 minutes per day in order to satisfy the body's requirement for vitamin D (19). The duration of sun exposure among Indonesian and Malaysian pregnant women in our study are 50 minutes and 4 minutes per day, respectively, which is lower than the recommended duration, thus might not be sufficient to detect a significant difference.

In line with a study conducted among Australian pregnant women (36), no significant association was found between sunscreen application and vitamin D deficiency in both Indonesian and Malaysian pregnant women. It is possible that sun avoidance behaviours in pregnant women who did not use sunscreen may have contributed to the non-significant findings in the present study. In addition, inadequate amount of sunscreen use may have little impact on the total blockage of UVB radiation (38).

In order to determine the shared modifiable environmental factors of vitamin D deficiency among Indonesian and Malaysian pregnant women, we conducted a multivariable analysis using the combined data from these two countries. After adjustment for potential confounding variables, we found that compliance to vitamin D intake according to the dietary recommendations of 15 mcg per day significantly reduces the risk of vitamin D deficiency among pregnant women in the combined cohort. However, it should be noted that the average intake of vitamin D among Indonesian and Malaysian pregnant women in our study was low and a majority of them did not achieve the recommended nutrient intakes. Given the gap between the recommended intakes of vitamin D and actual mean intakes in the present study, strategies to increase vitamin D intake are needed to prevent and control the epidemic of vitamin D deficiency among pregnant women in these two countries. As there are very few foods naturally containing vitamin D and these foods are infrequently consumed in Indonesia and Malaysia, intervention strategies such as dietary diversification may be less effective. A review by Yang et al. (20) demonstrated that fortification of edible oil with vitamin D at a level of 10 mcg/100 g could provide 3.9% to 21% of the Institute of Medicine Estimated Average Requirement (EAR) of vitamin D for adults in Southeast Asia. Thus, it is recommended that fortification of staple foods and condiments such as edible oil should be considered as one of the strategies to improve vitamin D status of pregnant women in Indonesia and Malaysia (20).

Results from the combined cohort also demonstrated that sun exposure of BSA of ≥27% significantly reduces the risk of vitamin D deficiency among pregnant women in the present study. Thus, previous recommendations by Holick (39) that vitamin D requirement of the body can be fulfilled by exposing hands, arms, and face (approximates to 19–27% of BSA) to the sunlight for 2 to 3 times a week may not be adequate to prevent vitamin D deficiency in tropical countries. Perampalam et al. (33) suggested that ≥27% of BSA should be exposed to the sun to induce an increase in vitamin D level. However, this suggestion may not be applicable to pregnant women wearing concealing clothing in tropical countries due to religion and cultural reasons. It is therefore recommended that women with concealing clothing should increase their duration of sun exposure to at least 64.5 minutes per day in order to satisfy their requirement for vitamin D as proposed by Judistiani et al. (19) in a study conducted among pregnant women in West Java, Indonesia.

There are several limitations should be taken into consideration. First, results from the present study should be interpreted with caution as different vitamin D assays were used across the two cohorts. However, our objective was to determine the shared environmental factors of maternal vitamin D status in two tropical countries. Hence, the discrepancies among the assays may not affect our conclusions, as the trends in 25(OH)D concentrations usually remain the same across different assays (40). Second, self-reported data on vitamin D intake and sun exposure may be subjected to recall and reporting bias.

In conclusion, vitamin D deficiency is prevalent among pregnant women living in tropical countries despite abundant sunshine. Dietary vitamin D intake of ≥15 mcg daily and exposure of ≥27% of BSA to the sunlight are protective against vitamin D deficiency among pregnant women living in tropical countries. Our study suggests that nutrition education and counseling for pregnant women in tropical countries should emphasise on vitamin D consumption and sun exposure for primary prevention of vitamin D deficiency.
